# Local Government Medical Officers and the Central Authorities

**Published:** 1911-09-09

**Authors:** 


### Public Health and Hygiene.

### LOCAL GOVERNMENT MEDICAL OFFICERS AND THE CENTRAL AUTHORITIES.

No one who has watched the growth of local government in this country can fail to have been struck by the extensive and increasingly important part played by the medical profession, both in an advisory and executive capacity as medical officers to the local authorities. The Poor Law Authority, the Health Authority, and the Education Authority have each of them their specially appointed medical officers, and both the local and central bodies have found it necessary in this way to equip themselves with a medical staff.

These medical services have grown up in truly British fashion—that is to say, they have not arisen as part of a clearly thought-out, theoretically consistent, and comprehensive scheme with a definite and intelligent purpose, but haphazard, as the pressure of circumstances urged, and in what is termed a "practical" manner.

The result has been what we see—not one body or authority concerning itself with things medical in public affairs, but almost all authorities concerning themselves tentatively, amateurishly, and even fearfully in matters of health as they come within the cognisance of the various authorities whose more proper domain has been encroached upon or has invaded the special province of State medicine. Thus, to mention only one of the results of this want of system, it would be necessary, if it were asked which is the government department of health in this country, to answer: The Local Government Board, the Board of Education, the Home Office, the Colonial Office, the Board of Trade, etc.

This anomaly in the organisation of Government departments has reflected itself in the confusion which reigns in our unorganised local medical services. The certifying factory surgeon, the police surgeon, the Post Office surgeon, the Poor Law medical officer, the public vaccinator, the school medical officer, the medical officer of health—all of them medical officers of State—have almost no other point in common.

The diffusion of interest and dissipation of effort in which such lack of system issues is extremely inimical to those great public interests which are identified with medicine. That the public gets an ample value in return for the money expended in public medical services will scarcely be questioned; but that that return is trivial in comparison with the results which would be possible were the medical services effectively organised is also unquestionable.

For the most part the different medical officers are voiceless. The medical officer of health almost alone can give publicity to his views, and even he is not infrequently constrained to do so through the medium of the Clerk to his Council. If the Local Government Board wishes to address the medical officer, over whom it exercises exceptional control, it does not write to him direct; it writes to somebody else—to the Town Clerk. If it desires information which it is alone within the power of the medical officer of health to supply, the Clerk is requested to communicate its desires to the medical officer. If the medical department of the Board of Education desires to communicate with the school medical officer, an Assistant Secretary of the Board writes to the Secretary of the Education Committee. Reports, circulars, memoranda issuing from the Government departments are sent, not to the medical officers of the local authorities whom they are intended to reach, but to the Clerks and Secretaries of these bodies. This circumlocution is the correct manner of conducting affairs where the Government departments are concerned in the medical aspects of local government.

Frequently these Clerks and Secretaries are able members of a learned profession, but not seldom they are men of indifferent education who have risen from the ranks of the local authorities' clerical staff. This idiosyncrasy of the Government departments of writing to C when they desire to communicate with B is apt to have a deleterious effect upon C—the Clerk or Secretary of the local authority. There is much smallness in human nature when the circumstances are favourable for its development. The Government department's selection of the clerical functionary as the exclusive mouthpiece of the local authority impresses the authority with his relative importance, with the result that the medical officer is apt to be listened to only if he succeeds in speaking through his official mouthpiece.

Town Clerks and Education Secretaries are continually circularising medical officers for technical information which will enable them to advise their authorities on matters on which medical men almost alone are competent to speak authoritatively. Advising the local authority upon hospital construction and equipment, disinfection, the organisation and administration of medical inspection and medical treatment of school children, the provision of dental clinics, the complex problems of school dentistry, physically and mentally defective schools, and others of a like highly technical character are matters which lie wholly outside the sphere of a clerical officer. The trained medical administrators, who in these matters are so frequently forestalled by officious amateurs, labour under great disadvantages in having to work badly conceived systems launched under the advice of a clerical official the functions of whose office have been misconceived.

The methods of modern scientific research and their application to the living social problems of today are a new wine which cannot be placed in the old bottles of antiquated, tape-bound and precedent-ridden customers of central and local clerical departments that have outgrown their chief and primary intention. If it is clear that medicine has a spacious future in the expanding rational life of the nation, it is equally clear that it must come into its own by the creation of a National Department of Health.

